# Does the use of proton pump inhibitors lead to an increased risk of dental implant failure? A systematic review and meta-analysis

**DOI:** 10.4317/medoral.27043

**Published:** 2025-05-27

**Authors:** Mingchun Tang, Zhimin Xie

**Affiliations:** 1 Department of Stomatology, Jili Hospital of Liuyang City, Hunan Province, China; 2 Department of Stomatology, Zhuzhou Hospital Affiliated to Xiangya School of Medicine, Central South University, Zhuzhou City, Hunan Province, China

## Abstract

**Background:**

Proton-pump inhibitors (PPI) are one of the commonly used medications for a variety of gastrointestinal disorders. Given the large population using PPI and the increased use of dental implants in recent times, it is pertinent to examine if PPI impacts implant outcomes. This systematic review examined the risk of implant failure amongst PPI users vs non-users.

**Material and Methods:**

PubMed, Embase, Scopus, and Web of Science literature databases were scouted for cohort or case-control studies comparing implant survival between PPI users vs non-users. Last date of the literature search was 30th October 2024.

**Results:**

We identified eight studies for inclusion. Both crude and adjusted data were pooled separately. Meta-analysis of crude data demonstrated that there was a statistically significant risk of implant failure in PPI users as compared to non-users (OR: 2.71 95% CI: 1.72, 4.29 I2 = 63%). These results failed to change on sensitivity analysis. Pooled analysis of adjusted data showed that PPI use may not independently predict implant failure (OR: 1.44 95% CI: 0.92, 2.24 I2 = 73%). Exclusion of one outlier study showed a significantly increased risk of implant failure with PPI use (OR: 1.71 95% CI: 1.17, 2.50 I2 = 42%).

**Conclusions:**

There may be a tendency for higher implant failure in patients using PPI. The lack of stability of the results on sensitivity analysis and non-significant associations noted with adjusted data preclude strong conclusions. There is a need for further high-quality studies to strengthen the available evidence.

** Key words:**Medications, implant survival, osteointegration, complication.

## Introduction

Dental implants have become one of the most common treatment modalities in the management of full and partial edentulism. A large body of evidence has demonstrated that dental-implant supported prostheses are among the safest and most viable treatment options for a large number of patients requiring prosthetic rehabilitation (1-3). Indeed, data suggests an exponential increase in the use of dental implants in the past few decades. In the USA alone, there has been a 14% increase in the use of dental implants every year and the numbers shall only increase in the coming decade (4). The survival rates of dental implants are usually high with 96.4% of implants surviving after 10 years (5). Nevertheless, given the high prevalence of dental implants, a significant number of implants fail. A number of risk factors have been identified in literature like smoking, comorbidities (diabetes, heart disease, osteoporosis), periodontitis, bruxism, cancer treatment, poor oral hygiene, prior infections, and reduced bone quality (6,7). While there are relatively few contraindications to implant placement, caution should be exercised especially in patients with comorbidities and taking concomitant medications (8). Research shows that a large number of commonly used drugs like anti-hypertensives, diuretics, corticosteroids, and anti-inflammatories can modulate bone metabolism (9-11) and therefore may potentially influence implant survival (12,13).

One important class of drug that deserves attention is proton-pump inhibitors (PPI). PPIs are among the most commonly prescribed drugs worldwide accounting for about 113 million prescriptions each year (14). Omeprazole is the most commonly used agent but others like pantoprazole, lansoprazole, and rabeprazole are also frequently prescribed (15). These agents primarily act by suppressing gastric acidity through the inhibition of H+/K+-ATPase pumps and are found to be more effective than H2-receptor antagonists for the management of peptic and duodenal ulcers (16). The action of PPI is not limited to gastric parietal cells alone as the transmembrane protein H+/K+-ATPase is also present on the surfaces of osteoclasts and inhibition of the same is thought to alter bone turnover and thereby implant osseointegration (17). Therefore, an important clinical query that arises is whether the use of PPI leads to an alteration in implant survival. This question has been previously examined by Vinnakota *et al* (18) in a systematic review and meta-analysis study of just three studies. Given the publication of newer studies, we hereby conducted an updated systematic review examining the impact of PPI use on dental implant failure.

## Material and Methods

We have reported the present study using the PRISMA guidelines (19) which also includes pre-registration of the protocol on PROSPERO for transparency. The registration number on PROSPERO was: CRD42024605707.

- Identification of studies

PubMed, Embase, Scopus, and Web of Science literature databases were explored online for identification of possible studies. Two reviewers were involved in the search which identified articles published between the inception of these databases to 30th October 2024. All authors agreed on the following search query which was developed in consultation with an experienced medical librarian: ((((((((Proton Pump Inhibitors) OR (Rabeprazole)) OR (Lansoprazole)) OR (Pantoprazole)) OR (Omeprazole)) OR (Dexlansoprazole)) OR (Esomeprazole)) OR (Ilaprazole)) AND (((((Dental implants) OR (dental implantation)) OR (Bone‐Anchored Prosthesis)) OR (Endosseous implants)) OR (Osseointegrated implants)). This query was used across all databases to search for relevant articles. An additional exploration of Google Scholar and reference lists of included studies completed the search strategy.

- Inclusion criteria

The PECOS inclusion criteria were used to identify possible studies. Details are as follows: 1. The population consisted of adult patients receiving dental implants for replacement of missing teeth. 2. Exposure variable was the use of PPI for any indication. 3. Comparison group was patients not using PPI. 4. Outcomes of interest included implant failure which was reported as either crude or adjusted data. 5. Only cohort and case-control studies were included.

The reviewers excluded studies which 1. Did not report separate outcomes for PPI use. 2. Studies not reporting implant failure. 3. Studies available only as abstracts. 4 Studies in non-English language. 5. Studies with duplicate data.

The selection process from the literature search followed a clear pre-defined process. To avoid duplicity of articles, we first excluded all duplicates electronically. The remaining studies were then examined by the two reviewers one by one by reading titles and abstracts only. In this initial step, non-relevant studies were removed and all remaining studies were downloaded. In the last step, the full texts were read and cross-checked against the inclusion criteria. When both reviewers were satisfied, the study was included in the review. Otherwise, any disagreements were resolved by consensus.

- Risk of bias and data management

The quality of the included studies was examined using the Newcastle-Ottawa scale (NOS). Both reviewers checked the individual articles against the queries of NOS which examines the selection of cohort, comparability of groups, and outcomes. Final scores were given after independent assessments by the reviewers which ranged from 0-9. Disagreements were resolved by consensus.

Two authors sourced information from the studies independently. It was later cross-checked for any errors. Data obtained for this review included: author name, publication year, study type, number of participants, number of implants, age and gender, smokers, number of PPI users, type of PPI used, type of implant placed, definition of implant failure, follow-up, and study results. We had initially planned in the protocol stage to examine the risk of implant failure and periimplantitis. However, since peri-implantitis was reported by just one study, we focused our review only on implant failure.

- Statistical analysis

Meta-analyses were performed using “Review Manager” (RevMan, version 5.3). The included studies reported either crude values or adjusted effect size or both for the effect of PPI on implant failure. For the meta-analysis, we extracted both crude and adjusted data and analyzed them separately. The outcomes were combined in the software to generate a pooled odds ratio (OR) with 95% confidence intervals (CI). The choice of meta-analysis model was random-effects. We also quantified the inter-study heterogeneity using the I2 index of the software. Values over 50% indicated substantial heterogeneity. A sensitivity analysis was performed by excluding one study at a time for each analysis to look for outliers. Since the number of studies was limited, funnel plots were used for publication bias.

## Results

- Search details

**Figure 1:**
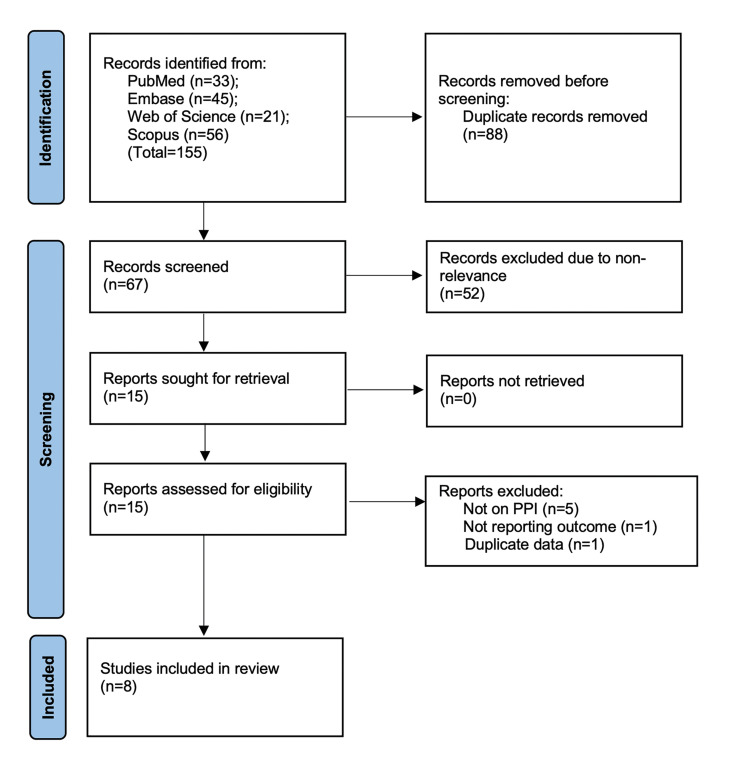
Study flowchart.

The number of studies identified in each database and the study selection process is presented in Fig. 1. The 155 studies identified initially underwent electronic deduplication. Herein, 88 studies were removed and 67 articles were further screened by the reviewers. Of these, only 15 were deemed relevant to the review after reading the title and abstract. Further analysis of full-texts led to the inclusion of eight studies (17,20-26).

- Baseline study details

All baseline data extracted by the reviewers from the studies can be found in Table 1. As noted, all studies were recent and published between 2017 and 2023. Based on study design, eight were retrospective cohort studies while one was a case-control study. The studies were conducted in the USA, Canada, Sweden, Israel and Turkey. The total number of participants in all eight studies was 5436. One study did not specify the exact number of implants placed. In the remaining seven studies, the total number of implants placed was 12,697. All studies included patients in the middle-aged or elderly age group. In one study, all smokers were excluded. In the remaining studies, the percentage of smokers ranged from 5.4 to 30.3%. Likewise, the exact number of PPI users was not reported by one cohort study while in the remaining studies, the percentage of PPI users varied from 4 to 35.6%. PPI use was either self-reported or identified from medical records in all studies. None of the studies were on a specific PPI and most included all PPIs. Follow-up varied from 12 to 94.8 months in the studies.

- Study quality

NOS scores awarded to the studies by the two reviewers by consensus are shown in Table 1. Three studies received a score of 9 indicating high quality. Two studies received a score of 8 while three studies received a score of 7. Studies receiving a score of 7 did not report adjusted data and hence were not awarded points for comparability of groups.

- Meta-analysis

**Figure 2:**
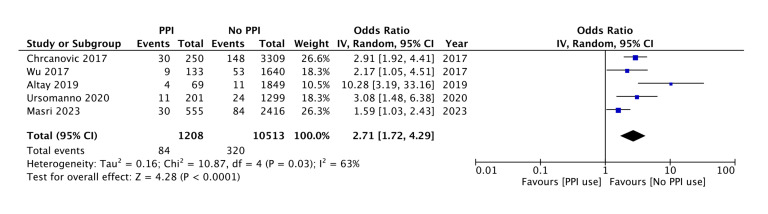
Meta-analysis examining the risk of implant failure with PPI using crude data.

Five of the eight studies reported crude data on implant failure while six of them reported multivariate adjusted data. The definition of implant failure used by the studies is shown in Table 2. Meta-analysis of crude data demonstrated that there was a statistically significant risk of implant failure in PPI users as compared to non-users (OR: 2.71 95% CI: 1.72, 4.29) (Fig. 2). We found significant heterogeneity in this meta-analysis as the I2 value was 63%. We also conducted a sensitivity analysis where we sequentially excluded studies from the meta-analysis only to find that the results remained statistically significant.

**Figure 3:**
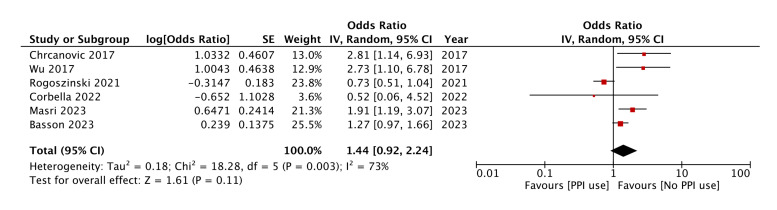
Meta-analysis examining the risk of implant failure with PPI using adjusted data.

**Figure 4:**
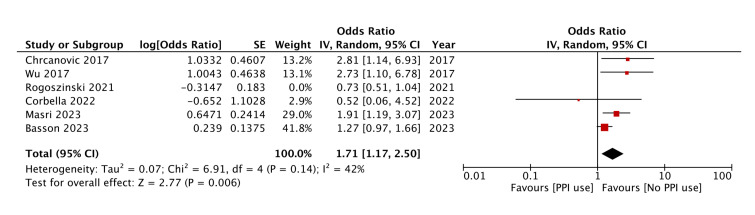
Meta-analysis examining the risk of implant failure with PPI using adjusted data after exclusion of Rogoszinski <italic>*et al*</italic> .

Meta-analysis of adjusted outcomes is shown in Fig. 3. Pooled analysis of adjusted data showed that PPI use may not independently predict implant failure (OR: 1.44 95% CI: 0.92, 2.24). This pooled analysis also showed significant heterogeneity (I2 value= 73%). When a sensitivity analysis was performed, it was noted that the exclusion of the study of Rogoszinski et a l(23) altered the significance of the effect size. The pooled analysis of the remaining studies indicated a significantly increased risk of implant failure with PPI use (OR: 1.71 95% CI: 1.17, 2.50). The heterogeneity was also reduced with an I2 value of 42% (Fig. 4).

## Discussion

The increased life expectancy in the modern world has also led to an aging population with an increasing prevalence of disabling diseases and the intake of related medications. Despite the high success rate of dental implants, the impact of systemic diseases and co-medications remains a matter of concern (12,13). Uncontrolled systemic conditions could have localized or systemic effects which may increase the breakdown of the peri-implant tissues leading to implant failure (27). Likewise, several long-term medications tend to have oral and or skeletal effects which may hamper implant survival (9-11). Ting *et al* (28) in a meta-analysis of eight studies have shown that patients taking anti-resorptive medications have a significantly higher risk of implant failure as compared to those not on anti-resorptive drugs. However, another study by Carr *et al* (29) has shown that patients on long-term bisphosphonates, anticonvulsants, calcium channel blockers, aspirin, anti-depressants, and corticosteroids did not have a higher risk of implant failure after adjustment of important covariates. Chappuis *et al* (12) have reviewed data from 17 studies examining the effects of non-steroidal anti-inflammatory drugs, selective serotonin reuptake inhibitors, bisphosphonates, antihypertensives, and PPI on dental implant failure. Of these, only selective serotonin reuptake inhibitors and PPI were found to increase the risk of implant failure, albeit with just two studies each for both drugs. Later, Vinnakota *et al* (18) also published their systematic review of three studies that showed that PPI use had a detrimental effect on implant survival. Nevertheless, their review could examine only crude data and did not analyze multiple covariate-adjusted data.

The present study builds upon the evidence generated by prior systematic reviews to provide a comprehensive analysis of the effect of PPI on the outcomes of dental implants. We conducted an updated literature search on multiple literature databases to include eight studies comparing dental implant survival between PPI users vs non-users. Importantly, we segregated crude and adjusted data for better interpretation of outcomes. Meta-analysis of crude data found that patients using PPI had a statistically significant 170% increased risk of implant failure. Moreover, significant results were noted in all five studies included in the meta-analysis and sensitivity analysis did not alter the results. Nevertheless, it should be noted that all included studies were retrospective and were susceptible to selection bias. Since it is impossible to randomize patients for PPI exposure, there ought to be baseline differences between the PPI and non-PPI groups some of which could impact implant survival. Literature shows that implant failure can be affected by systemic factors like smoking, diabetes, heart disease, osteoporosis, and cancer treatment (6,7). Localized factors like implant position, poor bone quality, bruxism, traumatic occlusion, excessive loading, and lack of oral hygiene can also affect failure rates (30). While the included studies reporting adjusted data may not have accounted for all known confounders, even partial adjustment based on available data presents better results as compared to crude data. Pooled analysis of adjusted data failed to demonstrate a statistically significant difference in the risk of implant failure between PPI users vs non-users. The forest plot shows that half of the included studies noted a significant association while another half found no significant impact of PPI on implant failure. During sensitivity analysis, we noted that there was one outlier study by Rogoszinski *et al* (23), the exclusion of which showed a 71% increased risk of implant failure with PPIs. However, the study of Rogoszinski *et al* (23) was one of the most robust studies included in the current review as they included only those patients who had a complete minimum follow-up of 60 months and a consistent exposure to PPI. The OR of 0.73 (95% CI: 0.51, 1.04) reported by Rogoszinski *et al* (23) in fact showed a tendency of a protective effect of PPI on implant survival. We believe that the lack of stability of the results on sensitivity analysis complicates the interpretation of evidence and does not conclusively establish the adverse effect of PPI on implant survival.

There have been other studies that have demonstrated contrasting effects of PPI on periodontal attachment and peri-implantitis. Chawla *et al* (31) in a retrospective study have found that PPI users tend to have a reduced incidence of higher probing depths indicating better soft tissue attachment as compared to non-users. Romadini *et al* (32) in a cross-sectional study found that PPI use was a protective factor that was significantly associated with reduced risk of peri-implantitis. Likewise, animal studies have also demonstrated variable effects of PPI on dental implant osseointegration. In a study on rats, Tetkin *et al* (33) did not find any difference in bone biochemical markers or implant torque values with omeprazole exposure for four weeks. On the other hand, Al Subaie *et al* (34) in a similar study design examined the effects of two weeks of exposure to omeprazole on bone healing and implant osseointegration in rats only to note reduced bone formation and decreased implant-bone contact with the use of PPI.

There are several possible mechanisms by which PPI can influence peri-implant tissues and bone turnover (31). Firstly, PPI causes alteration of gastric pH and gut microbiota which can in turn cause significant changes in oral microbial load (35,36). Changes in the number and distribution of pathogenic microorganisms can affect peri-implant health. PPI also influences bone metabolism by altering intestinal calcium absorption and serum calcium levels. This reduces the bioavailability of calcium for incorporation into bone, which causes compensatory hyperparathyroidism that increases bone turnover (37). Furthermore, osteoclasts also have H+/K+-ATPase pumps which can be inhibited by PPI at higher levels than those required for similar action in the gastric parietal cells. This causes a reduction in bone resorption capacity of osteoclasts and reduced bone turnover which may be detrimental to osteointegration (38). PPIs have also been associated with reduced iron absorption due to their gastric acid-suppressing action (39). Iron deficiency in turn has been associated with reduced bone health and therefore can affect implant survival (40). Nevertheless, these are just postulations and there is a need for further studies examining the oral effects of PPI particularly concerning peri-implant health.

There are certain limitations of our review. The low number of studies in the meta-analysis warrants caution in the interpretation of the results. Inconsistencies in the reporting of data further reduced the number of studies in each meta-analysis. Secondly, the review could only assess implant survival and not other relevant outcomes like the incidence of per-implantitis due to scarcity of data. Language restriction of the literature search is another drawback that could have excluded potential articles. Lastly, the high heterogeneity in the meta-analysis is also a cause of concern. We believe that variations in the type of implants, type, and duration of PPI exposure, and follow-up periods could have led to high heterogeneity. However, since the included study did not present data related to these factors a subgroup or meta-regression analysis was not possible.

The strength of the study is the fact that it is an updated and comprehensive meta-analysis examining the effects of PPI on implant survival. Unlike the previous review (18), we could include five new studies to present improved evidence. By conducting a meta-analysis of both adjusted and crude data and also performing a sensitivity analysis, we carefully scrutinized the available data to provide a detailed analysis.

## Conclusions

There may be a tendency for higher implant failure in patients using PPI. The lack of stability of the results on is a need for further high-quality studies to strengthen the available evidence.

## Figures and Tables

**Table 1:** Details of included studies.

Author	Location	Study type	Number of patients/ implants	Mean age	Male gender (%)	Smokers (%)	PPI users (%)	Type of PPI	Implant type	Follow-up (months)	NOS score
Wu 2017	Canada	RC	799/ 1773	56.6	46.2	23.4	7.3	NR	Nobel Biocare with TiUnite surface	17	9
Chrcanovic 2017	Sweden	RC	999/ 3559	60	47.9	26.3	6.7	All*	Mixed type	94.8	9
Altay 2019	Turkey	RC	592/ 1981	49	46.6	0	4	AII*	Solid-screw with sandblasted, acid-etched, or titanium plasma-sprayed surfaces	29	7
Ursomanno 2020	USA	RC	635/ 1480	NR	NR	NR	NR	NR	Straumann, Nobel Biocare, Astra Tech	31	7
Rogoszinski 2021	USA	RC	284/ 933	NR	93.2	28.5	34.6	NR	NR	60	9
Corbella 2022	Italy	RC	270/ 1118	58.5	43	23	9.6	NR	NR	62	8
Masri 2023	Israel	RC	687/ 2971	NR	38.2	5.4	17.3	AII*	Two-piece, internal hex, rough surface titanium	12	7
Basson 2023	Israel	CC	1170/ NR	53	48.7	30.3	35.6	NR	NR	12	8

*includes omeprazole, lansoprazole, dexlansoprazole, esomeprazole, pantoprazole, rabeprazole, ilaprazole. PPI, Proton pump inhibitor; NOS, Newcastle Ottawa Scale; NR, not reported; RC, retrospective cohort; CC, case control.

**Table 2:** Outcome definition reported by the studies.

Author	Definition of implant failure
Wu 2017	Implants with at least one of the following complications were defined as failures: (a) pain on function; (b) mobility; (c) radiographic bone loss equivalent to 1 = 2 of the implant length; (d) uncontrolled exudate; and (e) implant no longer in mouth
Chrcanovic 2017	Implant considered a failure if presenting signs and symptoms led to implant removal
Altay 2019	Condition necessitating implant removal prior to prosthetic loading due to advanced peri-implant bone loss and implant mobility
Ursomanno 2020	Implant that lost osteointegration and required removal
Rogoszinski 2021	Defined as a lack of osseointegration, implant mobility, or bone loss >1 mm/year evaluated at least 6 months after implant placement
Corbella 2022	Identified as dental implant that was lost spontaneously or removed due to failure of osseointegration
Masri 2023	Defined as implant removal within a period of up to 12 months from loading
Basson 2023	Not reported
